# The rates and measurement of adherence to acamprosate in randomised controlled clinical trials: A systematic review

**DOI:** 10.1371/journal.pone.0263350

**Published:** 2022-02-03

**Authors:** Kim Donoghue, Laura Hermann, Eileen Brobbin, Colin Drummond

**Affiliations:** 1 Department of Clinical, Educational and Health Psychology, University College London, London, United Kingdom; 2 National Addictions Centre, Addictions Department, Institute of Psychiatry, Psychology and Neuroscience, King’s College London, London, United Kingdom; 3 Institute of Clinical and Applied Health Research, University of Hull, Hull, United Kingdom; The University of Mississippi Medical Center, UNITED STATES

## Abstract

**Aim:**

The current research aims to systematically review the rates of adherence reported in randomised controlled clinical trials of acamprosate. It also sought to determine the reliability of the adherence monitoring and measurement methods used in these trials.

**Methods:**

The protocol for this review was pre-registered (PROSPERO: CRD42021230011). A search of the literature was conducted using OVID MEDLINE, Embase and PsycINFO from database inception to January 2021. Randomised controlled trials with a minimum sample size of 10 per treatment arm that compared the efficacy of acamprosate with placebo or other active medication in adults with a diagnosis of alcohol dependence were included. Data on rates of adherence, methods of measurement and monitoring of adherence was extracted from eligible studies independently in duplicate by two reviewers. A weighted mean adherence rate was calculated. The reliability of adherence monitoring methods was determined by calculating an adherence-assurance score based on the adherence monitoring method used. Risk of bias was assessed using the Cochrane Risk of Bias Tool.

**Results:**

Fifteen studies met the eligibility criteria involving 4,450 participants (2,480 participants in the placebo arms). A mean adherence rate of 88% (54.2–95.0%) was reported across studies that reported the percentage of medication taken. A mean adherence rate of 84.9% (56.4–91.3%) was reported for trials that reported the percentage of participants taking more than 80% of medication prescribed. There is low confidence in the methods used to monitor adherence with all clinical trials having a low adherence-assurance rating. Risk of bias was judged to be high for all included studies.

**Conclusions:**

Adherence to acamprosate in clinical trials can be poor with low confidence in the methods used to measure it. Adherence rates therefore might not be accurate, which has implications for determining the efficacy of acamprosate.

## Introduction

Alcohol consumption is a leading factor for disease burden worldwide, associated with 60 acute and chronic health conditions and the leading cause of premature death in those aged 15–49 years. In 2016, alcohol consumption was attributable to 2.8 million deaths worldwide [1]. Those requiring treatment for their alcohol use often undergo frequent episodes of withdrawal and resumption of drinking with up to 70% of people returning to drinking in the year following treatment [2].

Acamprosate is a safe, effective and cost-effective medication to help support relapse prevention [3]. Guidelines produced by the National Institute for Health and Care Excellence (NICE) recommend acamprosate as a first-line treatment, in conjunction with psychosocial therapy, to help support those who have completed alcohol withdrawal to remain alcohol free [4]. Acamprosate modulates the glutamatergic system and stabilises the imbalance between inhibitory (GABA) and excitatory (glutamate) neurotransmitters in the brain during alcohol withdrawal, whereby reducing the conditioned effect of alcohol and the negative reinforcement of the addiction [5–7].

Despite the therapeutic potential of acamprosate, poor adherence to the medication poses a problem for effectiveness in clinical practice. Adherence to a medication can be considered the extent to which a patient’s actions match the recommendations agreed with the prescriber [8]. Suboptimal outcomes may result from underdosing, overdosing or taking medication at incorrect intervals. Improved treatment outcomes for alcohol dependence are associated with better adherence to medications for alcohol relapse prevention [9, 10]. Medication adherence is a common problem across clinical care but is particularly an issue in chronic conditions and greater risk of poor adherence has been associated with those who misuse substances [11]. Since clinical trials offer a controlled environment where adherence can be monitored by research staff and payment may even be received for participation, medication non-adherence in clinical practice is likely to be substantially greater than in clinical trials.

The precise measurement of adherence in clinical trials is essential to accurately assess the efficacy of the medication under investigation. Methods for monitoring adherence in clinical trials include direct supervision, pill count, patient or clinician self-report, biochemical markers and electronic adherence monitoring. Pill count and patient self-report are often used to measure adherence in clinical trials, they can be inexpensive and place minimal burden on the participant. However, self-report may lead to an over-estimate of rates of adherence [12]. Electronic adherence monitoring that involves a medication bottle cap (e.g. Medication Events Monitoring System) that records when the bottle is opened is considered the gold-standard for clinical trials but is not feasible for routine clinical care [13].

The current research aims to systematically review the rates of adherence reported in randomised controlled clinical trials of acamprosate. It also sought to determine the reliability of the adherence monitoring and measurement methods used in these trials.

## Materials and method

The protocol for this systematic review was preregistered with the International Prospective Register of Systematic Reviews (PROSPERO: CRD42021230011). This paper complies with the PRISMA (Preferred Reporting Items for Systematic Reviews and Meta-analyses).

### Eligibility criteria

Studies were included if they were randomised controlled trials with a minimum sample size of 10 per treatment arm comparing the efficacy of acamprosate with placebo or other active medication for alcohol relapse prevention. Included studies were of adults (aged 18 or older) with a diagnosis of alcohol dependence (ICD or DSM). Studies were excluded if they used a cross-over or open label design or included pregnant women. The eligibility of trials was confirmed in line with the inclusion/exclusion criteria.

### Information source and search strategy

The electronic databases EMBASE, MEDLINE and PsycINFO (using the Ovid interface) were searched from database inception to the 3^rd^ January 2021, combing terms for alcohol dependence, acamprosate and randomised controlled trials (see S1 File for the full search strategy). Searches were limited to studies published in English.

### Study selection and data collection process

Search results were managed using Endnote and Microsoft Excel. Two reviewers (K.D. and L.H.) independently screened the titles and abstracts of all identified references. The full texts of potentially relevant articles were then screened independently in duplicate by the two reviewers. Any disagreement when screening titles, abstracts or full text documents was resolved by discussion between the two reviewers. Data from each relevant article was extracted independently in duplicate by the two reviewers using Microsoft Excel spreadsheets that had been pre-piloted. Discrepancies were discussed between the two reviewers and agreement reached. The Data extracted included; participant characteristics (number of participants in each study arm, age, gender, ethnicity), study characteristics (number of trial arms, length of treatment with acamprosate and comparator/placebo, country of the study, psychosocial intervention), medication adherence monitoring method (e.g. pill count), the frequency that adherence was measured (days), adherence rate (acamprosate and comparator/placebo), the measure of medication adherence used (e.g. % of prescribed medication taken), overall adherence rate (acamprosate and comparator/placebo).

### Outcome measures

The following outcomes were assessed; 1. Medication adherence rate, 2. Medication adherence monitoring method, 3. Frequency of adherence measurement (percentage of days that the monitoring was used), 4. Measure of medication adherence, 5. Length of treatment with medication (days).

### Data synthesis

Adherence rates for each trial was combined and weighted by sample size according to the adherence reporting method used. Where separate percentages were reported for active medication and placebo groups, the percentage of medication for the active medication group was taken. If separate percentages are reported for different sub-groups, for example type of psychological therapy received, all were included in the adherence rate calculations that were completed in Microsoft Excel. There was variation in the treatment length for the clinical trials included. Pearson’s correlation using IBM SPSS version 26 [14] was used to conduct a post hoc exploration of the impact of length of treatment on rate of adherence.

The method described by Swift et al. [15] was used to calculate an adherence-assurance score for the trials included in this review. All data was entered into Microsoft Word and calculated manually. To calculate the adherence-assurance score, adherence monitoring methods were assigned a monitoring confidence level which takes into consideration ability for circumvention; high/3 = supervision, medium/2 = Medication Events Monitoring System (MEMS), riboflavin or acamprosate levels, Low/1 = self-report, pill count, blister packs. The percentage of dosing days on which the monitoring method was used was calculated. Biological testing methods such as the presence of riboflavin were considered to provide confirmation of dosing on a single day. An adherence-assurance score was then calculated using the following formula; Adherence-assurance score = (monitoring confidence level) X (monitoring frequency).

Where multiple methods of adherence monitoring had been used concurrently a combined score was calculated by adding together the two adherence-assurance scores for the two methods. For trials that used two methods with the same confidence level (e.g. two low confidence methods such as pill count and self-report), no additional adherence assurance was allocated. Raw adherence-assurance scores were normalised to 100% and assigned an adherence-assurance rating of low (0–49%), medium (50–79%) or high (80–100%).

### Risk of bias

Eligible studies were assessed for risk of bias using the Cochrane Risk of Bias Tool [16], which included risk of bias arising from the randomisation process, deviations from the intended interventions, missing outcome data, measurement of the outcome and selection of the reported results Two reviewers assessed each relevant article independently with discrepancies resolved by discussion.

## Results

**Fig 1:**
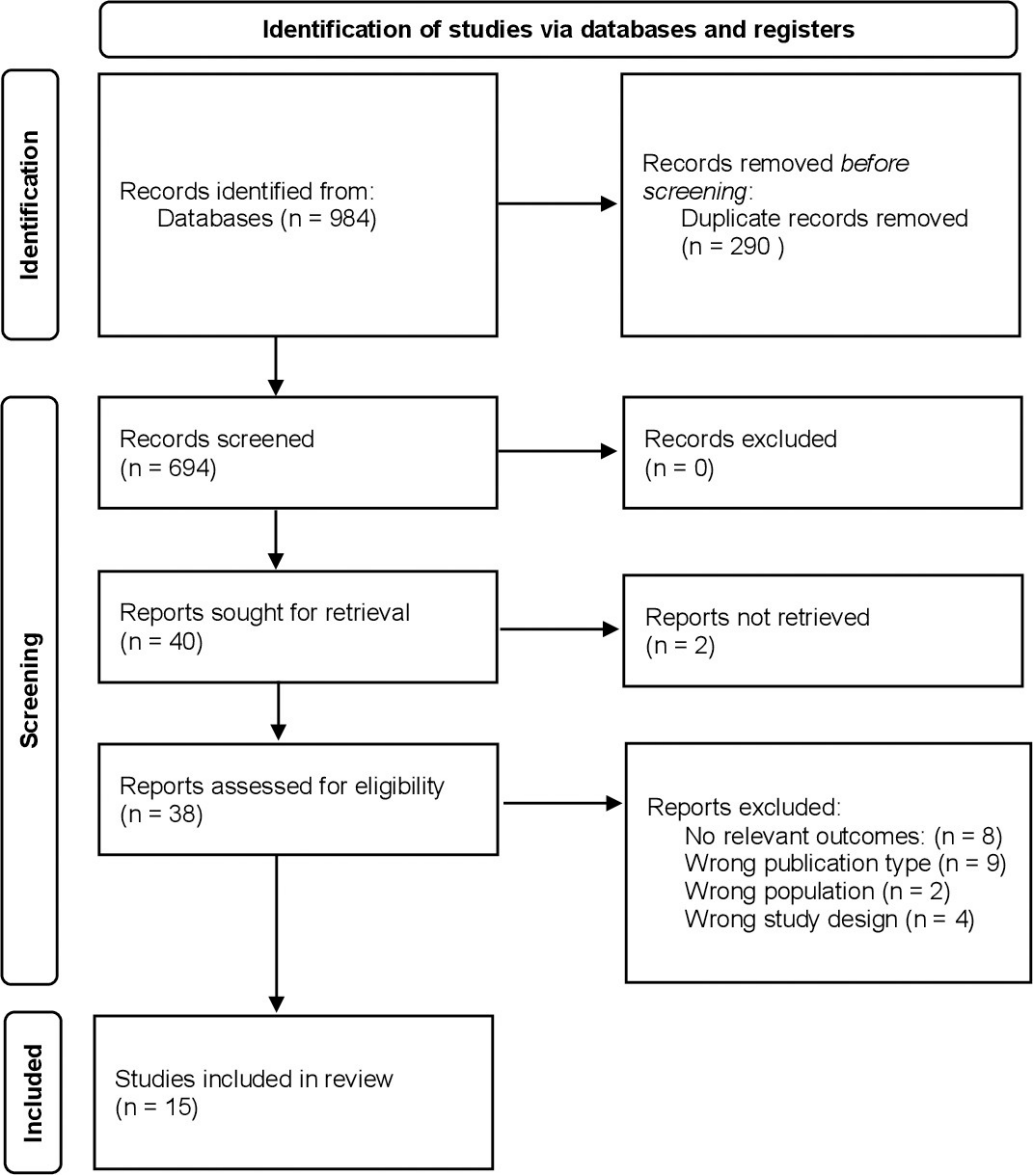
PRISMA flow diagram.

Fig 1 shows the results of the systematic search of the literature. A total of 15 studies were included involving 4,450 participants (2,480 participants in the placebo arms), the characteristics of these studies are presented in Table 1. The length of treatment with acamprosate ranged from 84 days to 365 days.

**Table 1:** Study characteristics.

Study	Country	Total N Acamprosate	Total N placebo	Participant age, mean (SD)	Participant gender, % male	Measure of adherence	Adherence rate Acamprosate	Adherence rate Placebo		Treatment length (days)	Risk of bias
Anton (2006)	USA	302 (MM = 152 and CBI = 150)	308 (MM = 153 and CBI = 156)	**MM** Acamprosate: 44.0 (SD 10.97 Placebo: 44.2 (SD 9.15) **CBI** Acamprosate: 45.4 (SD 10.32) Placebo: 43.2 (SD 9.74)	**MM** Acamprosate: 69.1% Placebo: 67.3% **CBI** Acamprosate: 70.9% Placebo: 70.5%	% prescribed meds taken	84.2%	NR		112	High
Berger (2013)	USA	51	49	Acamprosate: 46.6 (7.7) Placebo: 47.7 (8.5)	Acamprosate: 58.8% Placebo: 65.3%	% of prescribed meds taken	93.3%	91.6%		84	Some concerns
Besson (1998)	Switzerland	55	55	Acamprosate: 42.7 (NR) Placebo: 42.2 (NR)	Acamprosate: 83.6% Placebo: 76.4%	Not reported	Not reported but non-significant difference between groups noted except on the last study visit–those on placebo took significantly fewer tablets			360	High
Geerlings (1997)	Benelux i.e. The Netherlands, Belgium and Luxembourg	128	134	Acamprosate: 40.3 (9.2) Placebo: 41.7 (8.1)	Acamprosate: 76% Placebo: 76%	% medication taken	86%	88%		180	High
Gual (2001)	Spain	141	147	Acamprosate: 41.4 (9.01) Placebo: 40.7 (9.47)	Acamprosate: 80% Placebo: 79%	Average % medication taken per day	91.5%	97.8%		180	High
Higuchi (2015)	Japan	163	164	Acamprosate: 51.7 (12.4) Placebo: 53.1 (12.2)	Acamprosate: 86.5% Placebo: 88.4%	Not reported	Both groups included 7 pts whose adherence rate was below 70%, no intergroup differences			168	High
Kiefer (2003)	Germany	40	40	Acamprosate: 46.3 (7.7) Placebo: 45.6 (11.1)	Acamprosate: 75% Placebo: 68%	% prescribed medication taken	81.1% overall group specific not reported (Non-significant difference between groups noted			84	High
Mann (2013)	Germany	172	86	Acamprosate: 45.1 (8.5) Placebo: 46.6 (9.3)	Acamprosate: 80% Placebo: 78%	% prescribed medication	76.7%	73.5%		84	High
Mason (2006)	USA	2g/day = 258 3g/day = 83	260	Acamprosate: 2g/day = 44.9 (10.5), Acamprosate: 3g/day = 43.6 (8.9). Placebo = 44.5 (10.0)	Aamprosate 2g/day = 70% Acamprosate 3g/day = 71% Placebo = 64%	% prescribed medication	2g/day = 89.0%. 3g/day = 88.5%	92.6%		168	High
Morley (2006)	Australia	55	61	Acamprosate: 45.2 (9.2) Placebo: 42.4 (9.3)	Acamprosate: 76.4% Placebo: 71.7%	% taking 80% medication	56.4%	50.8%		84	High
Paille (1995)	France	361 (Dose 1.3g/day = 188 and Dose 2g/day = 173)	177	Acamprosate 1.3g/day: 43.7 (8.6) Acamprosate 2g/day: 43.3 (9.3) Placebo: 42.5 (8.9)	Acamprosate 1.3g/day: 77.7% Acamprosate 2g/day: 79.2% Placebo: 83.1%	% pills taken	**90 days:** Acp 1.3g/day = 80.3%, Acp 2g/day = 76.8%. **180 days**: ACP 1.3g/day = 70.5%, ACP 2g/day = 54.2%. **360 days:** ACP 1.3g/day = 54.9%, ACP 2g/day = 35.8%		**90 days** = 81.9%. **180 days**: 64.4%. **360 days** = 47.3%	365	High
Pelc (1997)	Belgium and France	126 (1332mg/day = 63 and 1998mg/day = 63)	62	NR	NR	% pills taken	95% of tablets not returned—overall, no group specific values reported			90	High
Sass (1996)	Germany	136	136	Acamprosate: 41.9 (8.4) Placebo: 40.5 (8.6)	Acamprosate: 75% Placebo: 80%	Not reported	Not reported but no difference between groups noted.			336	High
Tempesta (2000)	Italy	164	166	Acamprosate: 45.9 (11.33) Placebo: 45.9 (11.19)	Acamprosate: 84.8% Placebo: 80.7	Regular intake of study medication	76.9% - 84.5% ‘regular intake’, no group specific values reported, non-significant difference between groups noted			180	High
Wolwer (2011)	Germany	IBT: 124 TAU: 122	125	Acamprosate IBT: 45.1 (7.8) Acamprosate TAU: 45.7 (7.5) Placebo: 46.4 (7.7)	Acamprosate IBT: 75.8% Acamprosate TAU: 71.3% Placebo: 62.2%	% participants taking 80% or more of medication	91.3% overall			182	High

Nine studies reported the percentage of prescribed acamprosate taken with a weighted mean of 81.5% (range; 54.2% to 95%) [17–25]. Pearson’s correlation found no statistically significant correlation between the length of treatment with acamprosate and the percentage of prescribed medication taken (r = -0.308, p = 0.357). Two studies reported the proportion of participants taking at least 80% of prescribed acamprosate with a weighted mean of 84.9% of participants (range; 56.4% to 91.3%) [26, 27]. Three studies did not report adherence rates but stated that there were no differences between the acamprosate and placebo groups [28–30]. The final trial reported 76.9–84.5% of participants had regular intake of acamprosate during the trial with no differences between groups but no definition of regular intake was given [31]. The adherence assurance scores are reported in Table 2, all of which had a low adherence-assurance rating. All but one trial [29] used pill count to assess adherence to acamprosate, with ten of the trials relying solely on pill count. In addition to pill count, two trials utilised biological methods, Mason et al., [25] used plasma to monitor acamprosate adherence and Sass et al., [30] used urine analysis of acamprosate. Investigator assessment was used in addition to pill count by one trial [31] and a daily monitoring card was used by another trial [26]. A daily dosing card completed by participants was used as the only method of adherence assessment in in one trial [29]. All eligible studies were judged to have a high risk of bias, which was largely due to risk of bias arising from missing data (S1 Table).

**Table 2:** Adherence-assurance rating for acamprosate.

	Monitoring method A				Monitoring method B						
Study	Method	Adherence assurance score	Frequency (%)	Subscore	Method/confidence	Adherence assurance score	Frequency (%)	Subscore	Raw score (%)	Normal score (%)	Adherence assurance rating
**Acamprosate**											
Anton 2006	Pill count	1	100	100					100	33	Low
Berger 2013	Pill count	1	100	100					100	33	Low
Besson 1998	Pill count	1	100	100					100	33	Low
Geerlings 1997	Pill count	1	100	100					100	33	Low
Gual 2001	Not reported										
Higuchi 2015	Self-complete daily dosing diary	1	100	100					100	33	Low
Kiefer 2003	Pill count	1	100	100					100	33	Low
Mann 2013	Pill count	1	100	100					100	33	Low
Mason 2006	Pill count	1	100	100	Plasma acamprosate	2	2	4	104	35	Low
Morley 2006	PIll count and self report	1	100	100	Daily monitoring card	1	100	100	100	33	Low
Paille 1995	Pill count	1	100	100					100	33	Low
Pelc 1997	Pill count	1	100	100					100	33	Low
Sass 1996	Pill count	1	100	100	Urine-analysis of acamprosate levels	2	Not reported	Unknown	100	33	Low
Tempesta 2000	Pill count	1	100	100	Investigator assessment	1	100	100	100	33	Low
Wölwer 2011	Pill count	1	100	100					100	33	Low

## Discussion

Percentage of acamprosate taken during the clinical trials varied from 54.2% of prescribed medication taken to as high as 95%. However, the reliability of the methods used to measure adherence is low with the majority of trials relying on pill count. The risk of bias for the included trials was high, this was largely due to risk of bias arising from missing data. Dropout rates for the included trials was often high and it was unclear if this was taken into consideration when calculating adherence. Adherence rates may therefore have been inflated by only including those who completed the trial, which would be biased towards those who were adherent to acamprosate.

Since clinical trials offer a controlled environment where adherence can be monitored by research staff, medication non-adherence in usual clinical practice is likely to be greater. NICE [4] recommends that pharmacotherapies for alcohol relapse prevention are taken for at least 6 months, however, the length of time that these medications are taken often falls short of this. [32]. Therefore, service users may not be gaining the maximum benefit from acamprosate through poor medication adherence and not taking the medication for a sufficient period of time.

The impact of medication adherence on treatment effectiveness has been explored in research investigating the effect of adherence to acamprosate on alcohol outcomes. There is some evidence to suggest that non-adherence to acamprosate early in treatment is associated with poorer drinking outcomes [9, 33]. Effective methods of improving adherence to medications for alcohol relapse prevention are needed in both clinical trials and clinical practice. Simple interventions such as using text messages, dosette boxes and alarms to remind patients to take their medication are of value [34–36]. More complex psychosocial interventions such as Compliance Enhancement Therapy (CET) and Medical Management (MM) have been successfully used in clinical trials to support improved adherence to medication for those with alcohol dependence by promoting positive beliefs about medication and patient addressing concerns [17, 37]. Despite the successful inclusion of psychosocial interventions to enhance adherence in clinical trials, there has been little research into its application in a more typical clinical setting. Psychosocial interventions supporting adherence to medications for alcohol relapse prevention may not be directly transferable to clinical practice due to the burden on staff and costs of delivery [38]. Further research into how we can best support people completing treatment for alcohol dependence to take acamprosate as prescribed is needed.

This systematic review has identified a low confidence in the measures used to report adherence. A hierarchy from low to high confidence in the method used to monitor adherence to naltrexone has been proposed by Swift et al. [15]. The hierarchy, based on a patient’s ability to evade measurement of adherence, considered patient self-report and counting returned pills to have a “low” confidence. A “medium” confidence was assigned to Medication Events Monitoring System (MEMS) caps to electronically monitor pill bottle opening, or biomarkers such as the addition of riboflavin. Methods considered to have a “High” confidence included supervision of dosing, long-acting injectable preparations, or monitoring of the level of prescribed medication in the blood. Poor measurement and reporting of adherence to medications in clinical trials may lead to incorrect assertions about efficacy being made. Robust measurement is essential to ensure an accurate picture of medication adherence, which may be achieved using a combination of methods that are high/medium as well as low confidence. The implementation of standardised reporting of adherence rates and accurate, high confidence adherence measurement methods in clinical trials would assist with comparison of efficacy results across trials.

### Limitations

The results of this systematic review should be interpreted considering some limitations to the research. Studies were heterogeneous in their method, measurement and reporting of adherence to acamprosate making comparison between studies difficult and further subgroup analysis not possible. Adherence measures and methods were often poorly described in the trial papers making it difficult to determine the impact of missing data bias. The review only included papers published in English due to the language limitations of the authors. We were unable to assess publication bias in this systematic review. It is possible that publication bias could have led to an overestimation of the rates of adherence to acamprosate in clinical trials. There is an association between adherence to acamprosate and its efficacy and therefore unpublished trials with negative results may have had greater rates of non-adherence to acamprosate.

## Conclusions

The efficacy of acamprosate for alcohol relapse prevention is well documented. However, poor adherence to acamprosate may impact on its effectiveness in clinical practice. Pill count was the most common method of monitoring adherence, which has a low confidence. The method for measuring adherence was often poorly described and varied across studies identified in this review; harmonisation of these methods across studies would make comparison easier and results more transparent.

## Supporting information

S1 Checklist PRISMA 2020 for abstracts checklist. (DOCX)

S2 Checklist PRISMA 2020 checklist. (DOCX)

S1 File Search strategy. (PDF)

S1 Table Risk of bias. (DOCX)

## References

[1] Griswold MG, Fullman N, Hawley C, Arian N, Zimsen SR, Tymeson HD, et al. Alcohol use and burden for 195 countries and territories, 1990–2016: a systematic analysis for the Global Burden of Disease Study 2016. Lancet. 2018;392(10152):1015–35. doi: 10.1016/S0140-6736(18)31310-2 30146330 PMC6148333

[2] Hunt WA, Barnett LW, Branch LG. Relapse rates in addiction programs. J Clin Psychol. 1971;27(4):455–6. doi: 10.1002/1097-4679(197110)27:4&lt;455::aid-jclp2270270412&gt;3.0.co;2-r 5115648

[3] Jonas DE, Amick HR, Feltner C, Bobashev G, Thomas K, Wines R, et al. Pharmacotherapy for Adults With Alcohol Use Disorders in Outpatient Settings A Systematic Review and Meta-analysis. J Am Med Assoc. 2014;311:1889–900. doi: 10.1001/jama.2014.3628 24825644

[4] NCCMH. Alcohol-use Disorders: Diagnosis, Assessment and Management of Harmful Drinking and Alcohol Dependence. Leicester and London: The British Psychological Society and the Royal College of Psychiatrists.; 2011. 22624177

[5] Littleton J. Acamprosate in alcohol dependence: how does it work? Addict. 1995;90(9):1179–88. doi: 10.1046/j.1360-0443.1995.90911793.x 7580816

[6] Rösner S, Hackl-Herrwerth A, Leucht S, Lehert P, Vecchi S, Soyka M. Acamprosate for alcohol dependence. Cochrane Database Syst Rev. 2010;9. doi: 10.1002/14651858.CD004332.pub2 20824837 PMC12147086

[7] Cole J, Littleton J, Little H. Acamprosate, but not naltrexone, inhibits conditioned abstinence behaviour associated with repeated ethanol administration and exposure to a plus-maze. Psychopharmacol. 2000;147(4):403–11. 10.1007/s002130050009 10672634

[8] Nunes V NJ, O’Flynn N, Calvert N, Kuntze S, Smithson H, Benson J, et al. Clinical Guidelines and Evidence Review for Medicines Adherence: involving patients in decisions about prescribed medicines and supporting adherence. London: National Collaborating Centre for Primary Care and Royal College of General Practitioners.; 2009. 21834197

[9] Gueorguieva R, Wu R, Krystal JH, Donovan D, O’Malley SS. Temporal patterns of adherence to medications and behavioral treatment and their relationship to patient characteristics and treatment response. Addict Behav. 2013;38:2119–27. doi: 10.1016/j.addbeh.2013.01.024 23435273 PMC3595348

[10] Tolomeo S, Baldacchino A. Adherence to Prescribed Acamprosate in Alcohol Dependence and 1-Year Morbidities and Mortality: Utilizing a Data Linkage Methodology. Journal of clinical medicine. 2021;10(10):2102. doi: 10.3390/jcm10102102 34068243 PMC8153116

[11] Weiss RD. Adherence to pharmacotherapy in patients with alcohol and opioid dependence. Addict. 2004;99(11):1382–92. doi: 10.1111/j.1360-0443.2004.00884.x 15500591

[12] Kini V, Ho PM. Interventions to improve medication adherence: a review. J Am Med Assoc. 2018;320(23):2461–73. doi: 10.1001/jama.2018.19271 30561486

[13] Vrijens B, Urquhart J. Methods for measuring, enhancing, and accounting for medication adherence in clinical trials. Clin Pharmacol Ther. 2014;95(6):617–26. doi: 10.1038/clpt.2014.59 24739446

[14] Corp. I. IBM SPSS Statistics for Windows. 26.0 ed. Armonk, NY: IBM Corp; 2019.

[15] Swift R, Oslin DW, Alexander M, Forman R. Adherence monitoring in naltrexone pharmacotherapy trials: a systematic review. J Stud Alcohol Drugs. 2011;72(6):1012. doi: 10.15288/jsad.2011.72.1012 22051215 PMC9798465

[16] Schwingshackl L, Schünemann HJ, Meerpohl JJ. Improving the trustworthiness of findings from nutrition evidence syntheses: assessing risk of bias and rating the certainty of evidence. Eur J Nutr. 2020:1–11. doi: 10.1007/s00394-020-02464-1 33377996 PMC8354882

[17] Anton RF, O’Malley SS, Ciraulo DA, Cisler RA, Couper D, Donovan DM, et al. Combined pharmacotherapies and behavioral interventions for alcohol dependence. J Am Med Assoc. 2006;295(17):2003–17. doi: 10.1001/jama.295.17.2003 16670409

[18] Berger L, Fisher M, Brondino M, Bohn M, Gwyther R, Longo L, et al. Efficacy of acamprosate for alcohol dependence in a family medicine setting in the United States: a randomized, double-blind, placebo-controlled study. Alcohol Clin Exp Res. 2013;37(4):668–74. doi: 10.1111/acer.12010 23134193

[19] Geerlings P, Ansoms C, Van den Brink W. Acamprosate and prevention of relapse in alcoholics. Eur Addict Res. 1997;3(3):129–37.

[20] Gual A, Lehert P. Acamprosate during and after acute alcohol withdrawal: a double-blind placebo-controlled study in Spain. Alcohol Alcohol. 2001;36(5):413–8. doi: 10.1093/alcalc/36.5.413 11524307

[21] Kiefer F, Jahn H, Tarnaske T, Helwig H, Briken P, Holzbach R, et al. Comparing and combining naltrexone and acamprosate in relapse prevention of alcoholism: a double-blind, placebo-controlled study. Arch Gen Psychiatry. 2003;60(1):92. doi: 10.1001/archpsyc.60.1.92 12511176

[22] Mann K, Lemenager T, Hoffmann S, Reinhard I, Hermann D, Batra A, et al. Results of a double‐blind, placebo‐controlled pharmacotherapy trial in alcoholism conducted in Germany and comparison with the US COMBINE study. Addict Biol. 2013;18(6):937–46. doi: 10.1111/adb.12012 23231446

[23] Paille FM, Guelfi JD, Perkins AC, Royer RJ, Steru L, Parot P. Double-blind randomized multicentre trial of acamprosate in maintaining abstinence from alcohol. Alcohol Alcohol. 1995;30(2):239–47. 7662044

[24] Pelc I, Verbanck P, Le Bon O, Gavrilovic M, Lion K, Lehert P. Efficacy and safety of acamprosate in the treatment of detoxified alcohol-dependent patients. A 90-day placebo-controlled dose-finding study. Br J Psychiatry. 1997;171(1):73–7. doi: 10.1192/bjp.171.1.73 9328500

[25] Mason BJ, Goodman AM, Chabac S, Lehert P. Effect of oral acamprosate on abstinence in patients with alcohol dependence in a double-blind, placebo-controlled trial: the role of patient motivation. J Psychiatr Res. 2006;40(5):383–93. doi: 10.1016/j.jpsychires.2006.02.002 16546214

[26] Morley KC, Teesson M, Reid SC, Sannibale C, Thomson C, Phung N, et al. Naltrexone versus acamprosate in the treatment of alcohol dependence: a multi‐centre, randomized, double‐blind, placebo‐controlled trial. Addict. 2006;101(10):1451–62. doi: 10.1111/j.1360-0443.2006.01555.x 16968347

[27] Wölwer W, Frommann N, Jänner M, Franke PE, Scherbaum N, Lieb B, et al. The effects of combined acamprosate and integrative behaviour therapy in the outpatient treatment of alcohol dependence: a randomized controlled trial. Drug Alcohol Depend. 2011;118(2):417–22. doi: 10.1016/j.drugalcdep.2011.05.001 21621929

[28] Besson J, Aeby F, Kasas A, Lehert P, Potgieter A. Combined efficacy of acamprosate and disulfiram in the treatment of alcoholism: a controlled study. Alcohol Clin Exp Res. 1998;22(3):573–9. doi: 10.1111/j.1530-0277.1998.tb04295.x 9622434

[29] Higuchi S. Efficacy of acamprosate for the treatment of alcohol dependence long after recovery from withdrawal syndrome: a randomized, double-blind, placebo-controlled study conducted in Japan (Sunrise Study). J Clin Psychiatry. 2015;76(2):181–8. doi: 10.4088/JCP.13m08940 . 25742205

[30] Sass H, Soyka M, Mann K, Zieglgansberger W. Relapse prevention by acamprosate: results from a placebo-controlled study on alcohol dependence. Arch Gen Psychiatry. 1996;53(8):673. doi: 10.1001/archpsyc.1996.01830080023006 8694680

[31] Tempesta E, Janiri L, Bignamini A, Chabac S, Potgieter A. Acamprosate and relapse prevention in the treatment of alcohol dependence: a placebo-controlled study. Alcohol Alcohol. 2000;35(2):202–9. doi: 10.1093/alcalc/35.2.202 10787398

[32] Thompson A, Ashcroft DM, Owens L, van Staa TP, Pirmohamed M. Drug therapy for alcohol dependence in primary care in the UK: A Clinical Practice Research Datalink study. PLoS One. 2017;12(3):e0173272. doi: 10.1371/journal.pone.0173272 28319159 PMC5358741

[33] Koeter MW, van den Brink W, Lehert P. Effect of early and late compliance on the effectiveness of acamprosate in the treatment of alcohol dependence. J Subst Abuse Treat. 2010;39(3):218–26. doi: 10.1016/j.jsat.2010.06.002 20627222

[34] Nieuwlaat R, Wilczynski N, Navarro T, Hobson N, Jeffery R, Keepanasseril A, et al. Interventions for enhancing medication adherence. Cochrane Libr. 2014. doi: 10.1002/14651858.CD000011.pub4 25412402 PMC7263418

[35] Peng Y, Wang H, Fang Q, Xie L, Shu L, Sun W, et al. Effectiveness of mobile applications on medication adherence in adults with chronic diseases: a systematic review and meta-analysis. J Manag Care Sprc Pharm. 2020;26(4):550–61. doi: 10.18553/jmcp.2020.26.4.550 32223596 PMC10391210

[36] Stoner SA, Arenella PB, Hendershot CS. Randomized controlled trial of a mobile phone intervention for improving adherence to naltrexone for alcohol use disorders. PLoS One. 2015;10(4):e0124613. doi: 10.1371/journal.pone.0124613 25909320 PMC4409303

[37] Kranzler HR, Mueller T, Cornelius J, Pettinati HM, Moak D, Martin PR, et al. Sertraline treatment of co-occurring alcohol dependence and major depression. J Clin Psychopharmacol. 2006;26(1):13–20. doi: 10.1097/01.jcp.0000194620.61868.35 16415699

[38] Zullig LL, Deschodt M, Liska J, Bosworth HB, De Geest S. Moving from the trial to the real world: improving medication adherence using insights of implementation science. Annu Rev Pharmacol Toxicol. 2019;59:423–45. doi: 10.1146/annurev-pharmtox-010818-021348 30125127

