# Supplementary material for: The rates and measurement of adherence to acamprosate in randomised controlled clinical trials: A systematic review
Source: PLoS One. 2022 Feb 3;17(2):e0263350. doi: 10.1371/journal.pone.0263350 (PMC8812903; doi:10.1371/journal.pone.0263350)
Supplement: S1 File — (PDF) [file pone.0263350.s003.pdf]

The rate of adherence to the medication acamprosate for alcohol relapse prevention and the methods used to monitor and measure adherence to the medication in randomised controlled trials: A systematic review.

Search strategy

PsycInfo

1. Alcoholism/ or alcoholi*
2. Alcohol abuse/
3. Alcohol treatment/
4. Alcohol AND (abuse* OR dependen* OR disorder* OR addict* OR misuse OR problem)
5. Alcohol related disorder
6. OR/1-5
7. Acamprosate/ or acamprosate or campral
8. Treatment effectiveness evaluation/
9. Exp Treatment outcomes/
10. Psychotherapeutic outcomes/
11. Placebo/
12. Follow-up studies /
13. Placebo*.tw.
14. Random*.tw.
15. Comparative stud*.tw.
16. Randomi#ed controlled trial*.tw.
17. (clinical adj3 trial*).tw.
18. (Research adj3 design).tw.
19. (Evaluat* adj3 stud*).tw.
20. (Prospective* adj3 stud*).tw.
21. ((singl* OR doubl* OR trebl* or tripl*) adj3 (blind* OR mask*)).tw.
22. OR/8-21
23. AND 6, 7, 22

EMBASE

1. Alcoholism/ or alcoholi*
2. Alcohol abuse/
3. Alcohol AND (abuse* OR dependen* OR disorder* OR addict* OR misuse OR problem)
4. Alcohol related disorder
5. OR/1-4
6. Acamprosate/ or acamprosate or campral
7. Randomized controlled trial/
8. Controlled clinical study/
9. random$.ti,ab.
10. randomization/
11. intermethod comparison/
12. placebo.ti,ab.
13. (compare or compared or comparison).ti.

14. ((evaluated or evaluate or evaluating or assessed or assess) and (compare or compared or comparing or comparison)).ab.
15. (open adj label).ti,ab.
16. ((double or single or doubly or singly) adj (blind or blinded or blindly)).ti,ab.
17. double blind procedure/
18. parallel group$1.ti,ab.
19. (crossover or cross over).ti,ab.
20. ((assign$ or match or matched or allocation) adj5 (alternate or group$1 or intervention$1 or patient$1 or subject$1 or participant$1)).ti,ab.
21. (assigned or allocated).ti,ab.
22. (controlled adj7 (study or design or trial)).ti,ab.
23. (volunteer or volunteers).ti,ab.
24. human experiment/
25. trial.ti.
26. or/7-25
27. (random$ adj sampl$ adj7 (cross section$ or questionnaire$1 or survey$ or database$1)).ti,ab. not (comparative study/ or controlled study/ or randomi?ed controlled.ti,ab. or randomly assigned.ti,ab.)
28. Cross-sectional study/ not (randomized controlled trial/ or controlled clinical study/ or controlled study/ or randomi?ed controlled.ti,ab. or control group$1.ti,ab.)
29. (((case adj control$) and random$) not randomi?ed controlled).ti,ab.
30. (Systematic review not (trial or study)).ti.
31. (nonrandom$ not random$).ti,ab.
32. “Random field$”.ti,ab.
33. (random cluster adj3 sampl$).ti,ab.
34. (review.ab. and review.pt.) not trial.ti.
35. “we searched”.ab. and (review.ti. or review.pt.)
36. “update review”.ab.
37. (databases adj4 searched).ab.
38. (rat or rats or mouse or mice or swine or porcine or murine or sheep or lambs or pigs or piglets or rabbit or rabbits or cat or cats or dog or dogs or cattle or bovine or monkey or monkeys or trout or marmoset$1).ti. and animal experiment/
39. Animal experiment/ not (human experiment/ or human/)
40. or/27-39
41. 26 not 40
42. AND/5, 6, 41

Medline

1. Alcoholism/ or alcoholi*
2. Alcohol AND (abuse* OR dependen* OR disorder* OR addict* OR misuse OR problem)
3. Alcohol-related disorders/
4. OR/1-3
5. Acamprosate/ or acamprosate or campral

6. randomized controlled trial.pt.
7. controlled clinical trial.pt.
8. randomized.ab.
9. placebo.ab.
10. clinical trials as topic.sh.
11. randomly.ab.
12. trial.ti.

13. 1 or 2 or 3 or 4 or 5 or 6 or 7
14. exp animals/ not humans.sh.
15. 8 not 9
