# Supplementary material for: The rates and measurement of adherence to acamprosate in randomised controlled clinical trials: A systematic review
Source: PLoS One. 2022 Feb 3;17(2):e0263350. doi: 10.1371/journal.pone.0263350 (PMC8812903; doi:10.1371/journal.pone.0263350)
Supplement: S1 Table — (DOCX) [file pone.0263350.s004.docx]

S1 Table: Risk of bias

| Study ID | Risk of bias arising from the randomization process | Risk of bias due to deviations from the intended interventions | Risk of bias due to missing outcome data | Risk of bias in measurement of the outcome | Risk of bias in selection of the reported result | Overall |
| --- | --- | --- | --- | --- | --- | --- |
| Anton (2006) |  |  |  |  |  |  |
| Berger (2013) |  |  |  |  |  |  |
| Besson (1998) |  |  |  |  |  |  |
| Geerlings (1997) |  |  |  |  |  |  |
| Gual (2001) |  |  |  |  |  |  |
| Higuchi (2015) |  |  |  |  |  |  |
| Kiefer (2003) |  |  |  |  |  |  |
| Mann (2013) |  |  |  |  |  |  |
| Mason (2006) |  |  |  |  |  |  |
| Morley (2006) |  |  |  |  |  |  |
| Paille (1995) |  |  |  |  |  |  |
| Pelc (1997) |  |  |  |  |  |  |
| Sass (1996) |  |  |  |  |  |  |
| Tempesta (2000) |  |  |  |  |  |  |
| Wolwer (2011) |  |  |  |  |  |  |
